# Subsequent fertility after cesarean scar pregnancy: a retrospective analysis

**DOI:** 10.1186/s12884-023-05584-8

**Published:** 2023-05-11

**Authors:** Xiaoxiao Jin, Manman Liu, Panxi Zhang, Lingzhi Zheng, Feng Qi

**Affiliations:** Department of Gynecology and Obstetrics, Taizhou Hospital of Zhejiang Province, Zhejiang University, No. 150, Ximen St, Linhai, Zhejiang Province 317000 China

**Keywords:** Cesarean scar pregnancy, Fertility, Recurrent, Women’s health

## Abstract

**Background:**

Cesarean scar pregnancy (CSP) refers to the implantation and growth of the gestational sac at a uterine scarring site due to a previous cesarean section. The effects of CSP on subsequent fertility have emerged as a clinical issue of importance in gynecology and obstetrics in China owing to the increasing rate of cesarean section over the past 30 years in combination with the abolition of the national family planning policy, allowing for subsequent pregnancies. Therefore, we aimed to investigate the effects of CSP treatment on subsequent fertility and pregnancy outcomes.

**Methods:**

The study consecutively enrolled 499 women treated for CSP at Taizhou Hospital between January 2009 and December 2018. The study outcomes were the rate of secondary infertility and pregnancy outcomes. Clinical information was collected at the time of admission for CSP treatment. Information on subsequent fertility and pregnancy outcomes was collected via telephonic follow-up.

**Results:**

Among the 499 women who met the inclusion criteria for CSP, 48 were lost to follow-up. Most women (74.9%, 338/451) did not express the desire for a subsequent pregnancy after CSP treatment. Among the 113 women who initially desired a subsequent pregnancy, 62 finally abandoned fertility plans. Among the 51 women who pursued pregnancy, 48 pregnancies were recorded in 43 women, infertility secondary to CSP treatment was identified in 15.7% (8/51) of women, and 60.8% (31/51) of women achieved full-term pregnancy, with placenta accreta spectrum identified in two women, one requiring a hysterectomy during cesarean section due to massive bleeding. Among the 16 women treated with uterine artery embolization combined with uterine aspiration and 18 women treated by ultrasound-guided local lauromacrogol injection combined with uterine aspiration, a successful full-term pregnancy rate of 68.8% (11/16) and 88.9% (16/18), respectively, was achieved. There were five cases of recurrent CSP among all 76 pregnancies (6.6%).

**Conclusion:**

Over a long-term follow-up of women after CSP treatment, a high successful fertility rate was identified, with also an increased CSP recurrence rate. Uterine artery embolization combined with uterine aspiration and ultrasound-guided local lauromacrogol injection combined with uterine aspiration showed high rates of successful post-treatment fertility and pregnancy.

## Background

Cesarean scar pregnancy (CSP) refers to the implantation and growth of the gestational sac at the site of uterine scarring due to a previous cesarean section, and it has an incidence of approximately 1 per 2000 pregnancies globally [1, 2]. In China, the incidence of CSP has increased significantly owing to the increasing rate of cesarean section over the past 30 years in combination with the abolition of the national family planning policy, allowing for subsequent pregnancies [3, 4]. Accordingly, the diagnosis and treatment of CSP have emerged as clinical issues of importance in gynecology and obstetrics.

CSP can cause severe complications, such as massive vaginal bleeding, placenta accreta spectrum (PAS), uterine arteriovenous fistula, and uterine perforation [5–7]. The principle aim of CSP treatment is lesion ablation, followed by minimization of the impact on subsequent fertility and pregnancy. Currently, there are two main methods used for ablation of CSP: (1) medical therapies, such as intramuscular methotrexate (MTX) injection, local MTX injection, anhydrous alcohol, potassium chloride, or hypertonic glucose administration; (2) surgical approaches, including ultrasound-guided vacuum aspiration, hysteroscopic lesion resection, and laparoscopic, transvaginal, or transabdominal lesion resection; (3) and a combination of these treatments. Adjuvant treatment, such as uterine artery embolization (UAE), local lauromacrogol injection, cervical balloon compression, and high-intensity focused ultrasound, can help further manage CSP [8–13]. However, evaluation of the effectiveness of these treatments is limited as most women do not intend to conceive again after treatment and the impact on subsequent fertility is uncertain. Therefore, in this study, we aimed to evaluate the effects of CSP treatment on subsequent fertility and pregnancy outcomes among women treated for CSP at Taizhou Hospital over a 10-year period.

## Methods

### Study sample

Patients treated for CSP at Taizhou Hospital between January 2009 and December 2018 were eligible for this study. The inclusion criteria were as follows: (1) previous history of cesarean section; (2) a positive pregnancy test; (3) the following findings on transvaginal ultrasound: no visible gestational sac in the uterine cavity or cervical canal, presence of a gestational sac in the isthmus of the anterior uterine wall, with or without detection of a fetal heart, discontinuity in the anterior uterine wall on sagittal ultrasound views through the gestational sac, absence or thinning of the myometrium between the bladder and gestational sac, and high-speed and low-resistivity blood flow signals around the gestational sac on color Doppler flow imaging [14]; (4) gestational age less than 12 weeks; (5) no active inflammation; (6) no history of treatment for other diseases unrelated to CSP during the period of hospitalization; and (7) complete clinical data. The exclusion criteria were as follows: (1) gestational age greater than 12 weeks, (2) CSP combined with a heterotopic pregnancy, (3) and hysterectomy or sterilization performed at the same time as CSP treatment.

During the study period, 499 patients with CSP met our inclusion criteria. The following CSP treatments were performed: ultrasound-guided local MTX injection; UAE combined with uterine aspiration; ultrasound-guided uterine aspiration; ultrasound-guided local lauromacrogol injection combined with uterine aspiration; and transabdominal, laparoscopic, and hysteroscopic resection.

### Outcomes

The outcomes were secondary infertility, defined as absence of pregnancy over a period of 12 months without contraception, and pregnancy outcomes. Clinical information of the included women was collected at the time of admission for CSP treatment. Information on subsequent fertility and pregnancy outcomes was collected via telephonic follow-up by two of the authors (ML, PZ).

## Results

**Fig. 1:**
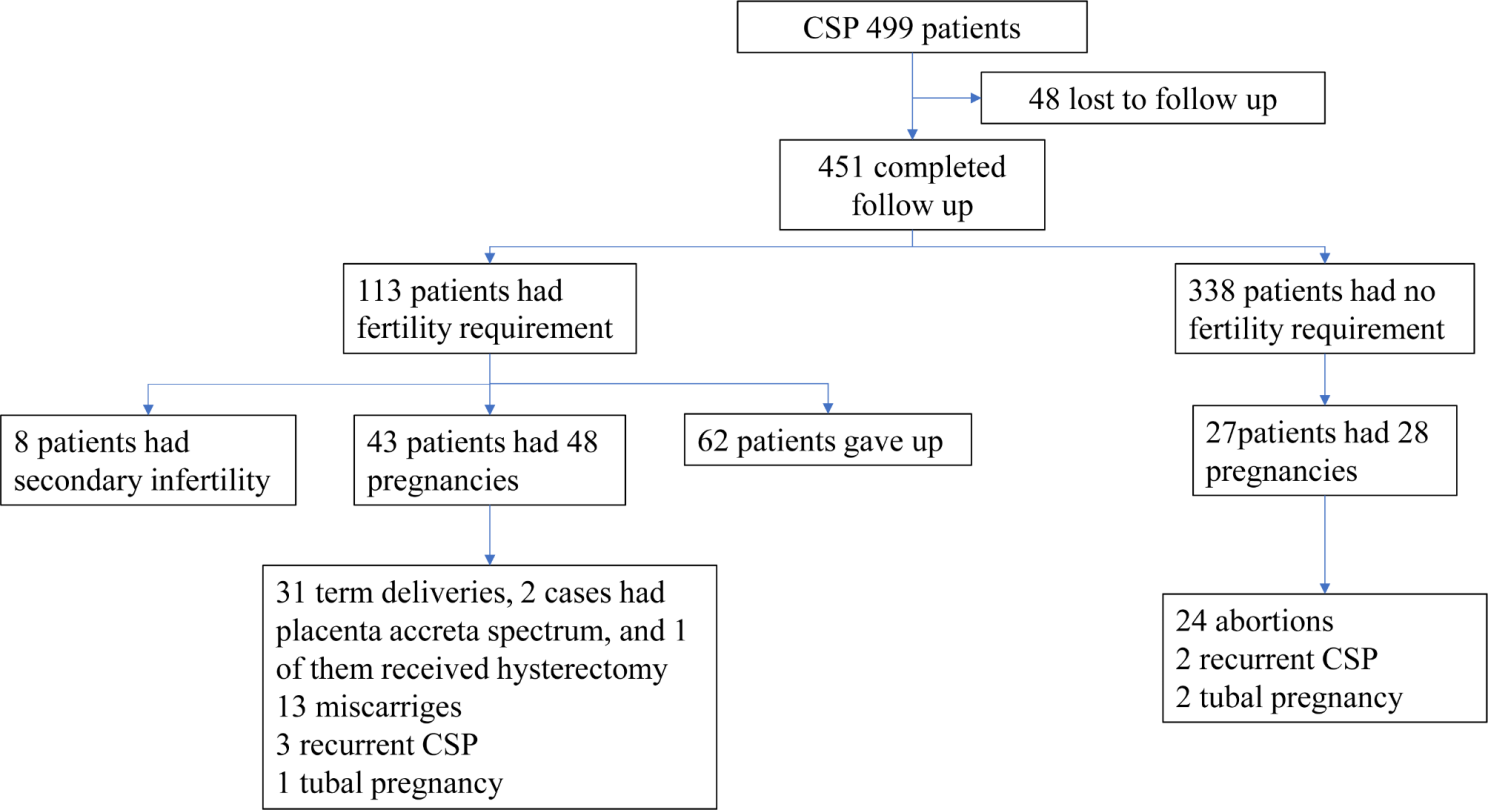
Distribution of fertility and pregnancy outcomes after previous cesarean scar pregnancy treatment

Among the 499 women who met the inclusion criteria for CSP, 48 were lost to follow-up. Among the remaining 451 women, 338 did not list fertility for a subsequent pregnancy as a goal. Among the remaining 113 women, 62 finally abandoned their fertility plans due to concerns of recurrent CSP, placenta accreta spectrum (PAS), and uterine rupture. Among the 51 women who desired a subsequent pregnancy, 48 pregnancies were recorded in 43 patients (Fig. 1). The pregnancies included: 31 full-term pregnancies with cesarean section delivery (two pregnancies with PAS, one pregnancy with massive bleeding during cesarean section, and one pregnancy with excessive bleeding during cesarean section requiring hysterectomy); 13 pregnancies with non-viable pregnancy outcomes, resulting in therapeutic abortion; three pregnancies with recurrent CSP; and one tubal pregnancy. Among the 51 women with fertility needs, there were eight cases of secondary infertility, all of which achieved a natural pregnancy without the aid of assisted reproduction methods before CSP. Lastly, among the 338 patients who had no fertility needs, 28 pregnancies were recorded in 27 women. Among these pregnancies, there were two tubal pregnancies, two recurrent CSPs, and 24 cases underwent artificial abortion after pregnancy. Therefore, the recurrence rate of CSP was 6.6% (5/76).

The following CSP treatments were used for 31 women who delivered at full-term: UAE combined with uterine aspiration in 11 cases, ultrasound-guided local lauromacrogol injection combined with uterine aspiration in 16 cases, ultrasound-guided local MTX injection in three cases, and ultrasound-guided uterine aspiration in one case. CSP treatment for eight cases of secondary infertility included UAE combined with uterine aspiration in five cases, ultrasound-guided local lauromacrogol injection combined with uterine aspiration in two cases, and ultrasound-guided local MTX injection in one case. Therefore, a successful pregnancy rate of 68.8% and 88.9% was achieved among the 16 women treated with UAE combined with uterine aspiration and the 18 women treated with ultrasound-guided local lauromacrogol injection combined with uterine aspiration, respectively. Among the five cases of recurrent CSP, UAE combined with uterine aspiration was used in two cases, ultrasound-guided local lauromacrogol injection combined with uterine aspiration was used in two cases, and ultrasound-guided uterine aspiration was used in one case. Among the three cases of tubal pregnancy treated for CSP, UAE and uterine aspiration were used in two cases and ultrasound-guided local lauromacrogol injection combined with uterine aspiration was used in one case. The characteristics of the women who had full-term pregnancy or ectopic pregnancy after CSP treatment are shown in Table 1.

**Table 1:** Characteristics of women with full-term pregnancy and ectopic pregnancy after cesarean scar pregnancy treatment

No.	Date	Age (years)	CS (no.)	GA (days)	Treatment	Interval (years)	Subsequent pregnancy
1	May 2013	30	1	47	Ultrasound-guided local injection of MTX	4	Full-term delivery (twin)
2	April 2015	26	1	52	Ultrasound-guided local injection of MTX	4	Full-term delivery
3	May 2016	32	2	43	Ultrasound-guided local injection of MTX	3	Full-term delivery
4	May 2015	28	1	58	UAE combined with uterine aspiration	6	Full-term delivery
5	December 2011	28	2	45	UAE combined with uterine aspiration	2	Tubal pregnancy
6	October 2014	32	1	51	UAE combined with uterine aspiration	2	Full-term delivery
7	August 2013	31	1	51	UAE combined with uterine aspiration	5	Full-term delivery
8	September 2013	30	1	79	UAE combined with uterine aspiration	4	Full-term delivery
9	June 2014	31	2	57	UAE combined with uterine aspiration	2	Full-term delivery
10	September 2014	27	1	38	UAE combined with uterine aspiration	1	Full-term delivery (hysterectomy due to PAS)
11	November 2014	29	1	38	UAE combined with uterine aspiration	3	Full-term delivery
12	November 2014	34	1	49	UAE combined with uterine aspiration	5	Full-term delivery
13	June 2015	33	2	65	UAE combined with uterine aspiration	3	Full-term delivery
14	October 2015	32	2	42	UAE combined with uterine aspiration	1	Recurrent CSP, received UAE and uterine aspiration
15	March 2016	29	1	40	UAE combined with uterine aspiration	1	Tubal pregnancy
16	April 2016	33	1	57	UAE combined with uterine aspiration	2	Full-term delivery
17	May 2016	32	1	41	UAE combined with uterine aspiration	3	Full-term delivery
18	May 2017	28	1	43	UAE combined with uterine aspiration	1	Recurrent CSP, received lauromacrogol and uterine aspiration
19	February 2014	24	1	87	Ultrasound-guided uterine aspiration	3	Full-term delivery
20	March 2017	30	1	58	Ultrasound-guided uterine aspiration	2	Recurrent CSP, received UAE and uterine aspiration
21	July 2016	36	1	47	Lauromacrogol + uterine aspiration	4	Full-term delivery
22	April 2017	35	1	39	Lauromacrogol + uterine aspiration	2	Full-term delivery
23	May 2018	32	2	45	Lauromacrogol + uterine aspiration	2	Full-term delivery
24	October 2017	32	1	53	Lauromacrogol + uterine aspiration	3	Full-term delivery
25	June 2017	35	1	60	Lauromacrogol + uterine aspiration	2	Full-term delivery
26	March 2017	30	1	47	Lauromacrogol + uterine aspiration	3	Full-term delivery
27	October 2017	28	1	57	Lauromacrogol + uterine aspiration	3	Full-term delivery (massive bleeding due to PAS)
28	March 2017	28	1	50	Lauromacrogol + uterine aspiration	1 and 3	Recurrent CSP, received lauromacrogol and uterine aspiration, and subsequent full-term delivery
29	July 2017	29	1	50	Lauromacrogol + uterine aspiration	1	Full-term delivery
30	May 2018	28	2	59	Lauromacrogol + uterine aspiration	3	Full-term delivery
31	December 2017	25	1	51	Lauromacrogol + uterine aspiration	2	Full-term delivery
32	February 2017	27	1	68	Lauromacrogol + uterine aspiration	2	Full-term delivery
33	March 2017	39	2	63	Lauromacrogol + uterine aspiration	2	Tubal pregnancy
34	July 2017	33	1	41	Lauromacrogol + uterine aspiration	2	Full-term delivery
35	June 2018	30	2	35	Lauromacrogol + uterine aspiration	2	Full-term delivery
36	November 2018	31	1	43	Lauromacrogol + uterine aspiration	1 and 3	lauromacrogol and uterine aspiration, and subsequent full-term delivery
37	February 2019	30	2	46	Lauromacrogol + uterine aspiration	2	Full-term delivery

CS, Cesarean sections; GA, Gestational age; MTX, Methotrexate; UAE, Uterine artery embolization; PAS, placenta accreta spectrum; CSP, Cesarean scar pregnancy

## Discussion

The relationship between CSP treatment and subsequent fertility and pregnancy outcomes has not been sufficiently evaluated to date. This may reflect the fact that most women who have experienced a CSP are fearful of recurrence, abandoning subsequent pregnancies despite the recurrence rate of CSP being relatively low [15, 16]. In our study sample, most women (74.9%) did not express the desire for a subsequent pregnancy after CSP treatment, with 54.9% (62/113) of women who initially intended a second pregnancy finally abandoning this goal due to concerns about recurrent CSP and its complications, such as PAS and uterine rupture.

Although women with a history of CSP are at risk of recurrent CSP and other serious maternal morbidities during the second pregnancy, those who retain reproductive function after CSP treatment can become pregnant again. In our study sample, the recurrence rate of CSP was 6.6% (5/76). Among the 51 women who intended to have a subsequent pregnancy, 31 (60.8%) achieved full-term pregnancy, with complications identified in two cases, namely PAS in two cases, with one case requiring hysterectomy during cesarean section due to massive bleeding. In a review of 73 women with CSP who retained their uterus after treatment, Sadeghi et al. [17] reported that 59 (81%) women became pregnant again, with CSP recurrence in 15 (25%) cases. In their follow-up fertility observation study of 189 CSP cases, Wang et al. [18] reported a recurrence rate of 15.6% among 32 women with a second pregnancy. In a recent single-center case series, eight women with a history of CSP had 10 repeat pregnancies, with CSP recurrence in four cases [19]. Nagi et al. [20] followed 21 women treated conservatively for CSP and reported a 5% recurrence rate of CSP during the second pregnancy. In their follow-up of 14 women treated conservatively for CSP, Seow et al. [21] reported that seven women achieved a subsequent pregnancy, four of which were intrauterine pregnancies delivered by cesarean section at 35–36 weeks of gestation. The other two pregnancies were complicated by PAS. One case was a triplet pregnancy, consisting of intrauterine twins and recurrent CSP, with cesarean section performed at 32 weeks of gestation with subsequent hysterectomy due to massive bleeding. The second case complicated by PAS was identified during cesarean section performed at 37 weeks of gestation. The remaining woman in the case series reported by Seow et al. became pregnant at 3 months after CSP treatment, which included curettage and cervical balloon compression; however, the patient sustained a spontaneous uterine rupture during pregnancy and died of hypovolemic shock with a stillbirth. Therefore, women who become pregnant again after CSP treatment should be informed of the risk for CSP recurrence and serious complications.

The lack of an optimal treatment strategy for CSP is partly due to the absence of sufficient evidence regarding the impact of different treatment options on subsequent fertility. A 5-year follow-up study of 10 women with a history of CSP attempting to become pregnant again, showed that six patients treated with UAE combined with dilatation and curettage succeeded in the birth of seven healthy babies [18]. A retrospective cohort study led by Chen et al. [22] reported a 23.7% rate of secondary infertility after high-intensity focused ultrasound or UAE combined with suction curettage under hysteroscopic guidance among 135 women with CSP. Another study reported that 79 women with CSP who received ultrasound-guided suction curettage tried to become pregnant again and 13 (16.5%) suffered from sterility [23]. A recent 2021 study reported a secondary infertility rate of 40% (16/40) in women treated with hysteroscopic therapy [24]. In our study sample, secondary infertility occurred in 8/51 (15.7%) women pursuing a pregnancy. Considering the CSP treatments received in these cases compared to CSP treatments for women who achieved a full-term pregnancy, a successful pregnancy rate of 68.8% and 88.9% was achieved among the 16 women treated with UAE combined with uterine aspiration and the 18 women treated with ultrasound-guided local lauromacrogol injection combined with uterine aspiration, respectively. Therefore, both of these CSP treatments may have a higher probability of successful subsequent pregnancy.

This study had some limitations. First, this was a retrospective observational study performed at a single center; therefore, the possibility of bias in CSP treatments, which would influence outcomes, cannot be denied. Second, as a high proportion of women in our study sample did not have a goal of pursuing a subsequent pregnancy after CSP treatment, our analysis of fertility and pregnancy outcomes is, in fact, based on a small sample (51 cases). Therefore, only a description of the association between CSP treatment and subsequent pregnancy and CSP recurrence was possible. Hence, further prospective studies with larger sample sizes are required for high-quality evidence regarding the effect of different CSP treatments on fertility and pregnancy outcomes.

## Conclusions

Over a long-term follow-up of women after CSP treatment, a high successful fertility rate was identified with also an increased CSP recurrence rate. Both UAE combined with uterine aspiration and ultrasound-guided local lauromacrogol injection combined with uterine aspiration showed higher rates of successful post-treatment fertility and pregnancy. Therefore, both methods may be safe options for women who would like to pursue a subsequent pregnancy. The findings of this study provide further insights into the optimal treatment for CSP in women who wish to preserve their fertility in the future. However, further studies are warranted to validate the study findings.

## Data Availability

The datasets used and/or analyzed during the current study are available from the corresponding author on reasonable request.

## Abbreviations

CSP: cesarean scar pregnancy
MTX: methotrexate
PAS: placenta accreta spectrum
UAE: uterine artery embolization
